# Vitamin B12 Reduces TDP-43 Toxicity by Alleviating Oxidative Stress and Mitochondrial Dysfunction

**DOI:** 10.3390/antiox11010082

**Published:** 2021-12-29

**Authors:** Yu-Mi Jeon, Younghwi Kwon, Shinrye Lee, Seyeon Kim, Myungjin Jo, Seongsoo Lee, Sang Ryong Kim, Kiyoung Kim, Hyung-Jun Kim

**Affiliations:** 1 Dementia Research Group, Korea Brain Research Institute, Daegu 41068, Korea; ekpd0345@kbri.re.kr (Y.-M.J.); yhkwon@kbri.re.kr (Y.K.); srlee@kbri.re.kr (S.L.); seyeon@kbri.re.kr (S.K.); jomj@kbri.re.kr (M.J.); 2 Department of Brain and Cognitive Sciences, DGIST, Daegu 41068, Korea; 3 Gwangju Center, Korea Basic Science Institute, Gwangju 61886, Korea; soolee@kbsi.re.kr; 4 School of Life Sciences, Kyungpook National University, Daegu 41566, Korea; srk75@knu.ac.kr; 5 BK21 FOUR KNU Creative BioResearch Group, Kyungpook National University, Daegu 41566, Korea; 6 Department of Medical Biotechnology, Soonchunhyang University, Asan 31538, Korea; 7 Department of Medical Sciences, Soonchunhyang University, Asan 31538, Korea

**Keywords:** TAR DNA-binding protein 43, amyotrophic lateral sclerosis, *Drosophila*, mitochondrial dysfunction, oxidative stress

## Abstract

TAR DNA-binding protein 43 (TDP-43) is a member of an evolutionarily conserved family of heterogeneous nuclear ribonucleoproteins that modulate multiple steps in RNA metabolic processes. Cytoplasmic aggregation of TDP-43 in affected neurons is a pathological hallmark of many neurodegenerative diseases, including amyotrophic lateral sclerosis (ALS), frontotemporal dementia (FTD), Alzheimer’s disease (AD), and limbic predominant age-related TDP-43 encephalopathy (LATE). Mislocalized and accumulated TDP-43 in the cytoplasm induces mitochondrial dysfunction and reactive oxidative species (ROS) production. Here, we show that TDP-43- and rotenone-induced neurotoxicity in the human neuronal cell line SH-SY5Y were attenuated by hydroxocobalamin (Hb, vitamin B_12_ analog) treatment. Although Hb did not affect the cytoplasmic accumulation of TDP-43, Hb attenuated TDP-43-induced toxicity by reducing oxidative stress and mitochondrial dysfunction. Moreover, a shortened lifespan and motility defects in TDP-43-expressing *Drosophila* were significantly mitigated by dietary treatment with hydroxocobalamin. Taken together, these findings suggest that oral intake of hydroxocobalamin may be a potential therapeutic intervention for TDP-43-associated proteinopathies.

## 1. Introduction

TDP-43 (TAR DNA-binding protein 43) is encoded by the TARDBP gene [1]; it predominantly localizes in the nucleus and regulates several RNA metabolic pathways, including RNA splicing, stability, and translation [2,3]. TDP-43 contains a nuclear localization sequence (NLS) and nuclear export signal (NES); therefore, it can shuttle between the cytoplasm and nucleus. Dysregulation of TDP-43 induces the cytoplasmic accumulation of TDP-43 [4,5]. Aggregated TDP-43 in the cytoplasm has been detected in many neurodegenerative diseases, including Alzheimer’s disease (AD) [6], frontotemporal dementia (FTLD) [7,8], limbic-predominant age-related TDP-43 encephalopathy (LATE) [9], and amyotrophic lateral sclerosis (ALS) [10,11,12]. Neurodegenerative diseases associated with TDP-43 pathology are designated ‘TDP-43 proteinopathy’ [13,14].

TDP-43 interacts with NF-κB, a key factor contributing to the inflammatory response, and activates NF-κB in microglia. Activated NF-κB induces the production of proinflammatory cytokines, such as IL-1β and TNF-α [15,16]. Furthermore, mitochondrial dysfunction is one of the major pathological features of TDP-43 proteinopathy. Previous studies showed that the overexpression of TDP-43 induces the mitochondrial accumulation of TDP-43 [17,18,19]. Accumulated TDP-43 in mitochondria disrupts mitochondrial functions, such as fission/fusion dynamics, ATP synthesis, and Ca^2+^ homeostasis [20,21]. In addition, mitochondrial cristae showed a ‘swollen’ or ‘degenerated’ form in EM images of the brains of patients with TDP-43 proteinopathy [18]. Moreover, TDP-43 interacts with mitochondrial proteins (such as VDAC1 and PHB2) that promote mitophagy [22]. In addition, mitochondrial accumulation of TDP-43 decreases the mitochondrial membrane potential, mitochondrial complex I activity, and unfolded protein response (UPR) and increases reactive oxygen species (ROS) production [23]. Prolonged ROS production causes oxidative stress in TDP-43-expressing cells, and it also exacerbates other cellular dysfunctions [24].

A recent clinical study indicated that methylcobalamin (vitamin B12) is a slightly effective for patients with early-stage ALS. This study suggested that methylcobalamin does not affect all patients with ALS, but it can extend survival and reduce disease progression in patients with early-stage ALS [25]. However, the role of vitamin B12 in TDP-43-induced neurotoxicity is not fully understood. Vitamin B12 (called cobalamin) is a water-soluble vitamin that plays an essential role in our body. Vitamin B12 has important roles in DNA synthesis, methylation and mitochondrial function [26]. In addition, vitamin B12 plays a critical role in myelination. Vitamin B12 deficiency leads to demyelination and reduced neurotrophic activity, and it causes multiple sclerosis [27]. Many studies have shown that vitamin B12 accelerates nerve innervation and recovery in individuals with multiple sclerosis (MS) [28,29]. Vitamin B12 is known as a redox-active cofactor and it functions as an ROS scavenger [30]. It reduces oxidative stress in the cytosol and mitochondria; thus, it prevents cellular death [31]. Additionally, vitamin B12 regulates oxidative stress-related inflammatory responses [31] and immune responses [32,33]. According to clinical research, inflammatory cytokine expression is higher in patients with Alzheimer’s disease presenting low vitamin B12 levels than in patients with normal vitamin B12 levels [34]. Furthermore, patients with Parkinson’s disease show lower vitamin B12 levels than patients with other diseases, such as progressive supranuclear palsy (PSP), multiple system atrophy (MSA), and mild cognitive impairment (MCI) [35].

Hydroxocobalamin (Hb) is a manufactured analog of the vitamin B12 (cyanocobalamin). The cyanide ligand of vitamin B12 is replaced by hydroxide in Hb [36]. There are two forms of active co-enzymes in vitamin B12, methylcobalamin and adenosylcobalamin. Hb serves as a precursor of methylcobalamin and adenosylcobalamin [36]. Previous studies indicate that both methylcobalamine and adenosylcobalamine are essential for the normal development and function of the central nervous system [37,38]. Since Hb is a precursor of two active vitamin B12 forms, it can function as both methylcobalamin and adenosylcobalamin [36,39]. Therefore, Hb is much more useful than other forms of vitamin B12 in the treatment of vitamin B12 deficiency.

In this paper, we found that TDP-43-induced neurotoxicity was rescued by hydroxocobalamin treatment. We found that hydroxocobalamin protects cells by regulating mitochondrial dysfunction without affecting cytoplasmic accumulation of TDP-43 aggregates. In addition, in the *Drosophila* disease model, the short lifespan and climbing defects induced by TDP-43 expression were significantly rescued by hydroxocobalamin treatment. Taken together, these findings suggest that vitamin B12 may be a potential therapeutic target for TDP-43-associated proteinopathies.

## 2. Materials and Methods

### 2.1. Reagents and Antibodies

The following reagents were purchased from the indicated companies: dimethyl sulfoxide (DMSO; MilliporeSigma, Burlington, MA, USA), rotenone (RT; MilliporeSigma), mifepristone (RU-486; MilliporeSigma), MG-132 (MilliporeSigma), PERK inhibitor (MilliporeSigma) and hydroxocobalamin hydrochloride (Hb; Santa Cruz Biotechnology, Hercules, CA, USA). The following antibodies were purchased from the indicated companies: TDP-43 (Proteintech, Rosemont, IL, USA), phospho-eIF2α (Cell Signaling Technology, Danvers, MA, USA), eIF2α (Cell Signaling Technology), α-tubulin (Cell Signaling Technology), COX-Ⅳ (Cell Signaling Technology), cleaved caspase-3 (Cell Signaling Technology), anti-mouse IgG (Cell Signaling Technology), anti-rabbit IgG (Cell Signaling Technology), phospho-PERK (Santa Cruz Biotechnology), and Lamin A/C (MilliporeSigma).

### 2.2. Cell Culture

The SH-SY5Y human neuroblastoma cell line (ATCC, Manassas, VA, USA) was maintained in Dulbecco’s modified Eagle’s medium (DMEM; Gibco, Waltham, MA, USA) supplemented with 10% heat-inactivated fetal bovine serum (FBS; Gibco) and 50 μg/mL penicillin–streptomycin (Gibco).

### 2.3. Lentiviral Transduction

Third-generation lentiviral constructs, including pLenti-C-mGFP-P2A-Puro (vector) and human WT TDP-43, were used to generate lentiviruses as previously described [40]. HEK293T cells were transiently transfected with pLenti-C-mGFP-P2A plasmids, a 3rd generation packaging system, using Lipofectamine 2000 diluted in serum-free CD293 medium (Life Technologies, Carlsbad, CA, USA) to generate lentiviral particles. The cell culture supernatants were collected 48 h later, filtered through a 0.45-mm filter prior to transduction, and particle titers were determined using a Lenti-XTM qRT–PCR titration kit (Clontech, San Francisco, CA, USA). Particles were aliquoted and stored at −80 °C until the experiment. SH-SY5Y cells were infected with GFP-tagged or GFP-tagged human WT TDP-43 (MOI 25) virus particles using 4 μg/mL polybrene (MilliporeSigma). Lentiviral particles were centrifuged at 900× *g* at 32 °C for 120 min and incubated overnight in a 37 °C CO_2_ incubator. The medium was changed to DMEM containing 10% FBS to avoid the toxicity of virus particles. Cells were harvested 2 or 3 days after infection.

### 2.4. Cytotoxicity Test

#### 2.4.1. CCK-8 Assay

A Cell Counting Kit-8 (CCK-8, Enzo Life Science, Farmingdale, NY, USA) was used to measure cytotoxicity according to the manufacturer’s instructions. Briefly, SH-SY5Y cells were seeded in 96-well plates and treated with rotenone or Hb for 24 h, in which DMSO was used as a negative control. CCK-8 solution (10 µL) was added to each well and was incubated at 37 °C for 2 h. The absorbance at 450 nm was measured using a microplate reader (Tecan, Mannedorf, Switzerland). Cell viability was reported as a percentage of the control. All experiments were performed in triplicate.

#### 2.4.2. Flow Cytometry Analysis of Annexin V-FITC/PI Staining

The numbers of necrotic and apoptotic cells were measured using an Annexin V-FITC apoptosis detection kit (BD Biosciences, San Jose, CA, USA) according to the manufacturer’s instructions. Briefly, rotenone-treated SH-SY5Y cells (1 × 10^6^ cells/well) were cultured with/without Hb pretreatment. Cells were then resuspended in 500 μL of binding buffer (10 mM HEPES, 140 mM NaCl, and 2.5 mM CaCl_2_ (pH 7.4) and incubated with 5 μL of FITC-conjugated Annexin V according to the manufacturer’s instructions. Subsequently, the cells were incubated for 15 min in the dark at 37 °C. After adding 4 μL of propidium iodide (necrotic cells), flow cytometry was performed within 1 h using a MoFlo Astrios instrument (Beckman Coulter, Brea, CA, USA).

### 2.5. Immunoblot Analysis

Total protein extracts for immunoblot analysis were prepared in Cell lysis buffer (Cell Signaling Technology) with freshly added protease and phosphatase inhibitor cocktail (Roche, Basel, Switzerland) by homogenizing cells. Next, an equal amount of protein lysates was separated on 4–12% Bis-Tris gels (Thermo Fisher Scientific, Waltham, MA, USA) or NuPAGE 3–8% Tris-Acetate gels (Thermo Fisher Scientific) and was transferred to a polyvinylidene difluoride (PVDF, Thermo Fisher Scientific) membrane. The membranes were blocked with TBS blocking buffer containing 5% skim milk (BD Biosciences) or 2% bovine serum albumin (BSA) for 1 h and incubated with primary antibodies for 12 h at 4 °C. Detection was carried out using an ECL-Plus kit (Amersham, Buckinghamshire, UK). Samples from three independent experiments were used, and the relative expression levels were determined using a Fusion-FX instrument (Viber Lourmat, Collegien, France).

### 2.6. Preparation of Soluble and Insoluble Cell Extracts

Proteins were fractioned according to their solubility by making some modifications to a protocol previously described. Cells were harvested, washed, and homogenized in lysis buffer containing protease and phosphatase inhibitor cocktails. The homogenate samples were centrifuged at 100,000× *g* for 30 min at 4 °C. Supernatants containing the soluble fractions were harvested. The resting pellets were further extracted with cell lysis buffer containing 2% SDS (Cell Signaling Technology), sonicated and then boiled at 95 °C for 5 min. The supernatant from this step was collected as an insoluble fraction.

### 2.7. Preparation of Nuclear and Cytoplasmic Extracts

For subcellular fractionation, cells were lysed by NE-PER nuclear and cytosolic ex-traction reagents kit (Thermo Fisher Scientific) according to the manufacturer’s procedures. Briefly, nuclear and cytoplasmic fractions were obtained in ice-cold CER I and CER II buffer by centrifugation at 16,000× *g* for 5 min at 4 °C. Cytosolic supernatants were harvested and frozen. The pellet was then resuspended by vortexing in ice-cold NER buff-er, and the extracts were centrifuged at 16,000× *g* for 10 min at 4 °C. Supernatants containing the nuclear extract were collected.

### 2.8. Mitochondrial Extraction

The mitochondrial fraction was obtained using a Mitochondria Isolation Kit (Thermo Fisher Scientific) according to the manufacturer’s protocol. Briefly, cell fractions were prepared in ice-cold mitochondrial A, B and C buffers through centrifugation at 12,000× *g* for 5 min at 4 °C. Supernatants containing the cytosolic extract were harvested, and pellets were solubilized in ice-cold 2% CHAPS in TBS buffer. After vortexing, samples were centrifuged. Supernatants containing mitochondrial extracts were harvested. The extracts were mixed with 4× Bolt LDS Sample buffer and 10× Bolt Sample Reducing Agent buffer and then boiled at 95 °C for 5 min.

### 2.9. Immunostaining

For immunocytochemistry, cells were fixed with 4% paraformaldehyde for 30 min at room temperature. Cells were washed three times with PBS and then blocked with 5% BSA for 1 h at room temperature. For cell permeability, the blocking buffer was made of PBS-T (0.3% Triton X-100; MilliporeSigma). The primary antibody diluted in PBS-T containing 1% BSA was incubated with cells overnight at 4 °C. The next day, cells were then washed three times with PBS and incubated with an Alexa Fluor-conjugated secondary antibody for 1 h at room temperature. Then, samples were mounted and observed using a fluorescence microscope (Nikon, Tokyo, Japan). Photomicrographs from three randomly chosen fields were obtained.

### 2.10. IncuCyte ZOOM^TM^ (IncuCyte^TM^ Cytotox Red Reagent to Detect Dead Cells)

Images were captured and analyzed to determine cell growth and proliferation using the IncuCyte ZOOM™ Live-Cell Imaging system (Sartorius, Göttingen, Germany) as previously described for monitoring the kinetics of proliferation and cytotoxicity of the cultured cells [41,42]. IncuCyte image assays quantify how rapidly the proportion of the area covered by cells increases with time as a function of the cell proliferation rate. SH-SY5Y cells were seeded into 96-well plates at a density of 0.8 × 10^4^ cells per well, and all cells were then infected with GFP-tagged or GFP-tagged human WT TDP-43. The Hb (10 µM) or vehicle (DMSO) group was transferred to the IncuCyte ZOOM™ apparatus, and incubations continued over 72 h, with images collected every 3 h. All images were analyzed for confluence (%).

### 2.11. Bioenergetics Measurements in Cultured Cells

The oxygen consumption rate (OCR) and extracellular acidification rate (ECAR) were measured in TDP-43-transfected SH-SY5Y cells using a Seahorse XF96 flux analyzer (Agilent, Santa Clara, CA, USA). Cell lines were seeded in 24 wells of an XF 96-well cell culture microplate at a density of 1.5 × 10^5^ cells/well in 200 μL of growth medium and incubated for 24 h at 37 °C with 5% CO_2_. After replacing the growth medium with 200 μL of XF assay medium supplemented with 5 mM glucose, 1 mM pyruvate and 4 mM glutamine, which was prewarmed at 37 °C, cells were degassed for 1 h before starting the assay procedure in a non-CO_2_ incubator. The OCR and ECAR were recorded at baseline, followed by sequential additions of 1 μM oligomycin, 2 μM FCCP, and 0.5 μM antimycin A plus 0.5 μM rotenone. Nonmitochondrial oxygen consumption (in the presence of antimycin A + rotenone) was subtracted from all OCR values, and outlier technical replicates outside the standard deviation of the mean were discarded for both ECAR and OCR. In SH-SY5Y cells, values were normalized to the mean protein level in each well measured using a BCA protein assay kit (Thermo Fisher Scientific).

### 2.12. Quantitative RT–PCR

The TDP-43-overexpressed cells were isolated RNA using the TRIzol Plus RNA Purification Kit (Invitrogen) according to the manufacturer’s instructions and 100 ng of RNA were synthesized into cDNA templates at 37 °C for 120 min using a High-Capacity cDNA Reverse Transcription kit (Thermo Fisher Scientific). Quantitative RT–PCR was conducted using the one-step SYBR^®^ PrimeScript^TM^ RT–PCR kit (Takara Bio Inc, Kusatsu, Shiga, Japan) according to the manufacturer’s instructions using an Applied Biosystems 7500 Real-Time PCR system (Applied Biosystems). Internal control was using 18S rRNA. The 2^−ΔΔCt^ method was used to analyzed relative changes in gene expression [43]. All primer sequences used in this study are found in Table S1.

### 2.13. Subcellular Fractionation and DNA Isolation

Cells were collected at 600× *g* for 3 min and resuspended in 500 μL of buffer containing 20 mM HEPES pH 7.4, 10 mM KCl, 2 mM MgCl_2_, 1 mM EDTA, 1 mM EGTA, and 1 mM DTT. The homogenates were incubated on ice for 20 min and centrifuged at 720× *g* for 5 min. Transfer supernatant (contain cytoplasm) into a fresh tube and keep on ice. Then, the supernatants were centrifuged at 10,000× *g* for 5 min for the isolation of cytoplasmic fraction. Finally, DNA was extracted from the supernatant or whole cell using the QIAamp DNA Blood Mini kit according to the manufacturer’s protocol (QIAGEN, Hilden, Germany) and 100 ng of DNA was used to quantitative RT-PCR. All primer sequences used in this study are found in Table S1.

### 2.14. Fly Strains

*Drosophila* stocks were maintained on standard cornmeal agar media at 24 °C unless indicated otherwise. UAS-TDP-43 was described previously [44]. All stocks were obtained from The Bloomington Stock Center (Indiana University, IN, USA).

### 2.15. Climbing and Lifespan Assays

TDP-43-overexpressed adult male flies (0 to 1 d old) were divided and moved into individual vials contained RU-486 (Mifepristone) or EtOH only fly growing media (n > 100). Flies were transferred into new media every two-day, dead flies were recorded. Adult climbing ability was evaluated using a previously described method [44].

### 2.16. Statistical Analyses

Data were analyzed using one-way ANOVA followed by a post hoc analysis as indicated, depending on the variables compared (GraphPad Prism Software, San Diego, CA, USA). Differences were considered significant when *p* < 0.05 and are indicated as follows: * *p* < 0.05; ** *p* < 0.01; *** *p* < 0.001; and n.s., not significant.

## 3. Results and Discussion

### 3.1. Hydroxocobalamin Attenuates Oxidative Stress-Induced Neuronal Cell Death

**Figure 1:**
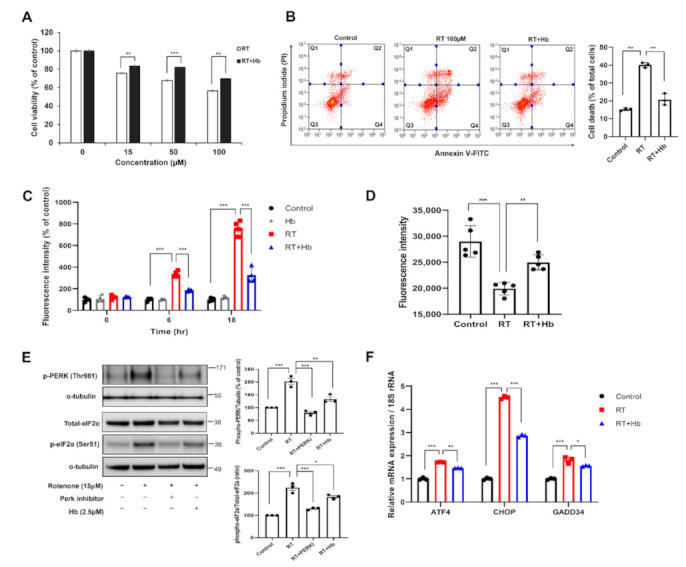
Hydroxocobalamin suppresses rotenone-induced PERK-eIF2α phosphorylation in SH-SY5Y cells. (**A**,**B**) SH-SY5Y cells were pretreated with 2.5 μM Hb for 30 min and then subsequently cultured with various concentrations of rotenone for 24 h. (**A**) Cell viability was measured using a CCK-8 assay. Data are presented as the mean percentages of untreated controls ± SD of three independent experiments. *** *p* < 0.001 (unpaired Student’s *t* test). (**B**) Representative flow cytometry plots obtained using Annexin V-FITC/PI staining for cell death analysis. Each cytogram was divided into four quadrants. Q1: Populations of Annexin-V-negative and PI-positive cells considered necrotic cells. Q2: Both Annexin-V-positive and PI-positive cells were marked and represent a later stage of apoptosis and necrosis. Q3: Negative for both Annexin-V and PI, indicating cells considered viable. Q4: Cells positive for Annexin-V and negative for PI that are presumed to be undergoing an early stage of apoptosis. The percentage of cell death is expressed as the sum of quadrants Q1, 2 and 4. Data are presented as the means ± SD of three independent experiments. Statistical analyses were performed using one-way ANOVA followed by Tukey’s multiple comparison test (*** *p* < 0.001). (**C**–**E**) SH-SY5Y cells were preincubated with Hb (2.5 μM) for 30 min and subsequently treated with rotenone (15 µM) for 18 h. (**C**) Intracellular ROS production was detected using the dye DCF-DA, and the results are reported as a percentage of the control by setting the sample fluorescence value of untreated cells to 100%. Data are presented as the means ± SD of three independent experiments. *** *p* < 0.001 (one-way ANOVA with Tukey’s multiple comparison test). (**D**) TMRE staining and analysis using a fluorescent plate reader. Error bars represent the mean values ± SD of independent experiments. The significance of differences was determined using one-way ANOVA with Tukey’s multiple comparison test (** *p* < 0.01 and *** *p* < 0.001). (**E**) Immunoblot analysis showing changes in the protein and phosphorylation levels of ER stress-related proteins following rotenone stimulation and treatment with Hb. For quantification, α-tubulin served as the internal control. The data are presented as the means ± SD of three independent experiments. ** *p* < 0.01 and *** *p* < 0.001 (one-way ANOVA with Tukey’s multiple comparison test). (**F**) SH-SY5Y cells pretreated with Hb (2.5 µM) were treated with rotenone (15 µM) for 8 h, and then real-time PCR was performed to assess the expression of ER stress-related genes. The mRNA levels are presented as the means ± SD of three independent experiments. 18S rRNA was used for normalization. * *p* < 0.05, ** *p* < 0.01, and *** *p* < 0.001 (one-way ANOVA with Tukey’s multiple comparison test).

Rotenone is a natural pesticide that increases ROS production and ER stress by impairing the mitochondrial electron transport system [45,46,47,48]. Chronic exposure to rotenone is known to increase the risk of Parkinson’s disease (PD), and oral rotenone administration has been widely used to model PD in rodents [49,50]. We performed a CCK-8 cell viability assay to investigate the effect of Hb on rotenone-induced cell death. The CCK-8 assay showed that rotenone-induced cytotoxicity was significantly alleviated by Hb treatment in SH-SY5Y cells (Figure 1A). We also confirmed this result using a different experimental approach based on flow cytometry using Annexin V (apoptotic cell death) and PI staining (necrotic cell death). The flow cytometry analysis showed that rotenone-induced cell death was substantially mitigated by Hb (Figure 1B). Pretreatment with Hb (2.5 µM) significantly reduced both apoptotic cell death (16.14 ± 4.00%) and necrotic cell death (4.60 ± 1.26%). These results indicate that Hb exerts a protective effect on ROS-induced neurotoxicity.

Next, we tried to determine whether Hb exerts a protective effect against other types of stress, such as proteotoxic stress. MG132, a potent ubiquitin proteasome system inhibitor, induces the death of SH-SY5Y cells. However, Hb treatment did not affect MG132-induced cytotoxicity in SH-SY5Y cells (Supplementary Material Figure S1B).

Previous studies showed that the inhibition of mitochondrial complex I activity induces ROS production, ATP depletion and the loss of the mitochondrial membrane potential [48,51,52]. Moreover, the increase in ROS production induces ER stress-mediated neurotoxicity. Therefore, we tried to determine whether ROS generation induced by rotenone is modulated by Hb. First, we investigated the effect of Hb on rotenone-induced intracellular ROS production using the redox-sensitive dye 2′,7′-dichlorofluorescin diacetate (DCF-DA). The rotenone-treated group showed a significant time-dependent increase in ROS production compared to the control group. The intracellular ROS level was significantly reduced in the group preincubated with Hb for 30 min and then treated with rotenone (Figure 1C). No significant difference was observed between cells treated with Hb alone and the control cells. Rather, ROS production was confirmed to be slightly lower after 24 h of exposure to rotenone (data not shown). We also investigated whether the mitochondrial membrane potential was altered by rotenone using tetramethylrhodamine ethyl ester (TMRE) staining. The depletion of the mitochondrial membrane potential is one of the characteristics of mitochondrial dysfunction. Not surprisingly, the mitochondrial membrane potential of rotenone-treated cells was significantly reduced compared to that of control cells. However, Pretreatment with Hb (2.5 μM) significantly mitigated the rotenone-induced reduction in the mitochondrial membrane potential (Figure 1D).

According to previous studies, rotenone induces apparent ER stress and increases the phosphorylation of PERK, PKR, and eIF2α [46,48,53]. We analyzed the levels of ER stress markers using immunoblotting to determine whether Hb alleviated rotenone-induced ER stress. Hb significantly reduced the rotenone-induced phosphorylation of PERK (Thr981) and eIF2α (Ser51) (Figure 1E). The activity of the PERK signaling pathway was inhibited by pretreating cells with a PERK inhibitor (GSK2606414) for 30 min followed by an incubation with rotenone for 24 h. In contrast, total eIF2α and PERK protein levels were not altered in rotenone- or rotenone/Hb-treated SH-SY5Y cells. Phosphorylation of eIF2α promotes the transcription of *ATF4*/*CHOP*/*GADD34,* and upregulation of these genes plays an essential role in cell death [54,55,56]. Thus, we measured the levels of *ATF4*/*CHOP*/*GADD34* transcripts in rotenone- or rotenone/Hb-cotreated SH-SY5Y cells using real-time PCR. We observed increased levels of the *ATF4, CHOP,* and *GADD34* transcripts in cells exposed to rotenone, and their levels were significantly decreased by Hb treatment (Figure 1F). Taken together, Hb suppresses rotenone-induced ER stress, thereby mitigating neurotoxicity. However, the neurotoxicity of MG132 was not significantly affected by Hb. These results suggest that Hb does not attenuate general proteotoxic stress, such as an impaired UPS.

### 3.2. Hydroxocobalamin Mitigates TDP-43-Induced Neurotoxicity

**Figure 2:**
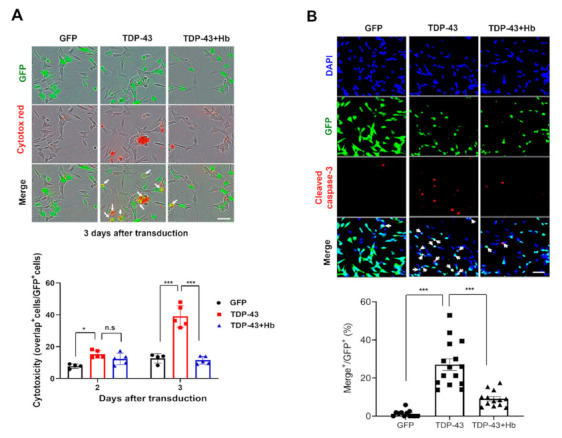
Hydroxocobalamin attenuates TDP-43-induced apoptosis in SH-SY5Y cells. (**A**) Hb (10 μM) was administered the day after SH-SY5Y cells were infected with GFP or GFP-tagged human WT TDP-43 lentivirus. Cells were stained with Cytotox Red (dead) and imaged every 3 h using the IncuCyte live cell imaging system. Double-fluorescent (green and red) cells were counted and quantified. Data are presented as the means ± SD of three independent experiments. * *p* < 0.05, *** *p* < 0.001, and n.s., not significant (one-way ANOVA with Tukey’s multiple comparison test). Scale bar, 50 μm. (**B**) SH-SY5Y cells were infected with a lentivirus encoding GFP or GFP-tagged human WT TDP-43 and cultured for 2 days, followed by treatment with Hb (10 μM) for 24 h (the total virus infection period was 3 days). Immunocytochemistry was subsequently performed to detect cleaved caspase-3 (red) and DAPI (nuclear, blue). Arrowheads indicate colocalization of GFP-positive cells and cleaved caspase-3, and Hb inhibited TDP-43-induced apoptotic death in SH-SY5Y cells. The ratio of cleaved caspase-3-positive cells in the control (GFP) or GFP-tagged human WT TDP-43-expressing cells was quantified. Error bars represent the mean values ± SD of three independent experiments. The significance of differences was determined using one-way ANOVA with Bonferroni’s multiple comparison test (*** *p* < 0.001). Scale bars, 50 μm.

We used a lentiviral system to express GFP-tagged human TDP-43 or GFP and determine whether TDP-43 induces cytotoxicity in SH-SY5Y cells. At 2 or 3 days after infection, transfected cells were subjected to immunocytochemical and biochemical analyses. A live-cell imaging system capable of real-time monitoring of cell viability was used to examine the effect of Hb on TDP-43-induced neuronal toxicity. SH-SY5Y cells were plated in 96-well plates and infected with either GFP-tagged TDP-43 or GFP lentiviral particles. Dead cells were labeled with Incucyte^®^ Cytotox Red dye, and cells were imaged every 3 h. The day after lentivirus infection, 10 μM Hb was administered, and cell death induced by TDP-43 was observed during concurrent treatment with Hb until the 3rd day of infection. TDP-43-induced neurotoxicity was observed on Day 3 after transduction, and Hb treatment significantly attenuated TDP-43-induced cell death (Figure 2A). No significant difference in cell death was observed in the group treated with Hb alone compared with the control group (data not shown).

Furthermore, we measured the level of cleaved caspase-3 (a marker of apoptosis) [57]. SH-SY5Y cells were infected with GFP or GFP-tagged human WT TDP-43 lentivirus, cultured for 2 days, and then treated with Hb (10 μM) for 1 day for a total experimental period of 3 days. Consistently, the number of cleaved caspase-3-positive SH-SY5Y cells was significantly increased on Day 3 of TDP-43 infection compared to the control cells. TDP-43-induced cell death was strongly suppressed by Hb treatment (Figure 2B).

**Figure 3:**
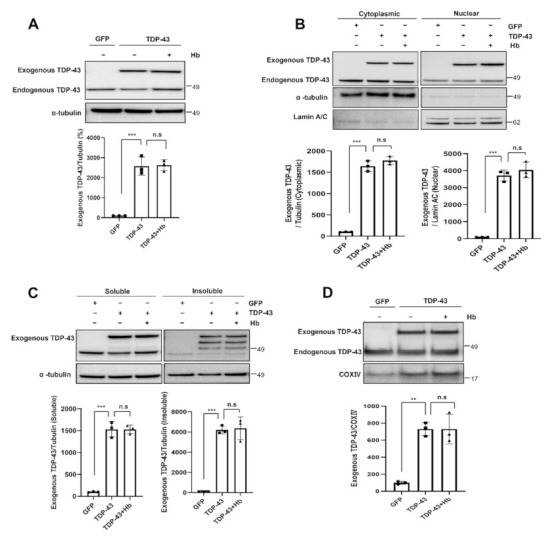
Hydroxocobalamin did not affect the aggregation and mislocalization of TDP-43. In the immunoblot analysis, cells were infected with the GFP- or GFP-tagged human WT TDP-43 lentivirus and grown for 2 days. The day before harvest, cells were treated with Hb (10 µM) for 24 h. (**A**) Cell lysates were prepared, and samples were immunoblotted with anti-TDP-43 antibodies. Hb treatment did not affect the TDP-43 protein levels. (**B**) Cytoplasmic and nuclear localization of exogenous TDP-43 in neuronal cells treated with Hb. Bar diagrams showing cytoplasmic and nuclear TDP-43 levels normalized to α-tubulin or Lamin A/C, respectively, are shown. Data are presented as the means ± SD of three independent experiments. *** *p* < 0.001 and n.s., not significant (one-way ANOVA with Tukey’s multiple comparison test). (**C**) Immunoblot analysis of soluble and insoluble exogenous TDP-43 protein levels in TDP-43-expressing cells treated with Hb. Error bars represent the mean values ± SD of three independent experiments. *** *p* < 0.001 and n.s., not significant (one-way ANOVA with Tukey’s multiple comparison test). (**D**) Immunoblot analysis showing the localization of the exogenous TDP-43 protein in the mitochondrial fraction. Normalization was performed using COX IV as a marker of purified mitochondria. Data are presented as the means ± SD of three independent experiments. Statistical analyses were performed using one-way ANOVA followed by Tukey’s multiple comparison test. ** *p* < 0.01, *** *p* < 0.001, and n.s., not significant.

We performed immunoblot analyses to elucidate the effect of Hb on TDP-43 protein levels. The level of the exogenous TDP-43 protein was not affected by Hb treatment. Therefore, Hb does not appear to exert a direct effect on the TDP-43 protein level (Figure 3A). Next, we investigated the effect of Hb on the cytoplasmic mislocalization of TDP-43 in neuronal cells. Interestingly, Hb treatment did not affect the levels of cytoplasmic and nuclear TDP-43 (Figure 3B). In addition, we examined whether the solubility of the exogenous TDP-43 protein was affected by Hb in neuronal cells. Hb treatment also did not affect the levels of soluble and insoluble exogenous TDP-43 in TDP-43-expressing cells (Figure 3C).

Recent studies have reported that the inhibition of TDP-43 mitochondrial localization blocks mitochondrial dysfunction and TDP-43-induced neurotoxicity [17,58]. We performed an immunoblot analysis to determine exogenous TDP-43 localization in the mitochondrial fraction and to evaluate whether Hb altered the mitochondrial localization of TDP-43. Exogenous TDP-43 was highly located in the mitochondrial fraction. Hb treatment did not affect the mitochondrial localization of TDP-43 in neuronal cells (Figure 3D). Thus, these results suggest that Hb had no effect on the localization and aggregation of the exogenous TDP-43 protein in the cytoplasm. In addition, we also found that the level of the endogenous TDP-43 protein was not altered by Hb treatment (analytical data not shown).

### 3.3. Hydroxocobalamin Suppresses TDP-43-Induced ER Stress

**Figure 4:**
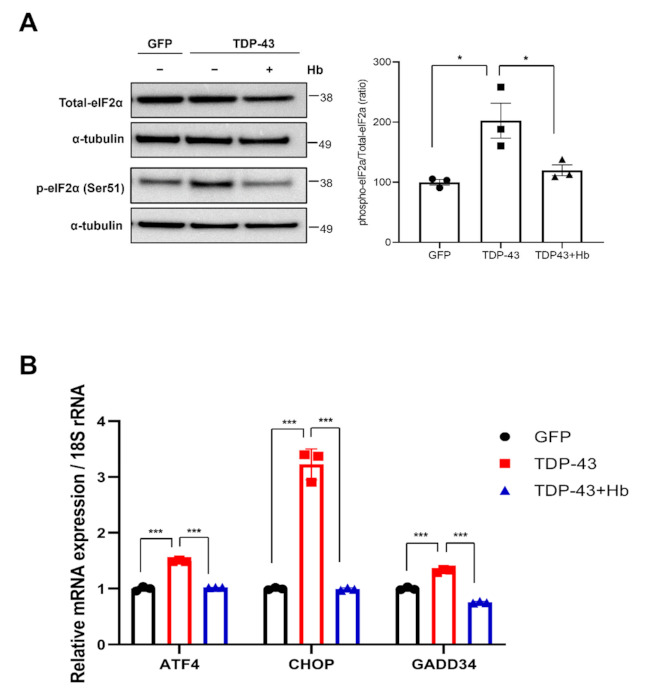
Hydroxocobalamin mitigates the TDP-43-induced unfolded protein response in neuronal cells. SH-SY5Y cells were infected with GFP or GFP-tagged human WT TDP-43 lentiviruses and treated with Hb (10 µM) simultaneously for 1 day. (**A**) Immunoblot analysis of eIF2α phosphorylation in TDP-43-expressing cells. Hb treatment significantly reduced the increase in eIF2α phosphorylation induced by TDP-43. Quantification was performed in three independent experiments and normalized to α-tubulin. Means ± SD. * *p* < 0.05 (one-way ANOVA with Tukey’s multiple comparison test). (**B**) Transcript levels of ER stress-related genes, including *ATF4*, *CHOP*, and *GADD34*, were detected using real-time PCR. 18S rRNA was used as an internal control. Error bars represent the mean values ± SD of three independent experiments. The significance of differences was determined using one-way ANOVA with Tukey’s multiple comparison test (*** *p* < 0.001).

Numerous studies have revealed that increases in PERK phosphorylation and eIF2α phosphorylation are associated with several neurodegenerative diseases [44,59,60]. Prevention of eIF2α phosphorylation might be an effective therapeutic approach for neurodegenerative diseases [61,62]. Therefore, we examined whether Hb modulated the eIF2α signaling pathway in TDP-43-expressing neuronal cells. SH-SY5Y cells were infected with GFP- or GFP-tagged TDP-43-encoding lentiviruses and treated with Hb for 24 h immediately after transduction. TDP-43 expression increased the level of eIF2a phosphorylation. Additionally, the increased eIF2α phosphorylation was significantly reduced by the Hb treatment (Figure 4A). Furthermore, we examined the transcript levels of downstream target genes in TDP-43-expressing cells using real-time PCR analysis to confirm the activation of ER stress by eIF2α phosphorylation. Transcript levels of target genes, including *ATF4*, *CHOP*, and *GADD34,* were significantly increased in TDP-43-infected cells compared to the controls, and Hb treatment significantly reduced the expression levels of those genes (Figure 4B). We also assessed the transcript levels of the ER stress response genes under Hb posttreatment conditions. For RT–PCR, cells were infected with TDP-43-GFP- or GFP-containing lentivirus. Infected cells were treated with Hb 24 h after transduction, and these cells were harvested 24 h after Hb treatment (2 days after transduction). Similar to the Hb pretreatment, the upregulation of *ATF4*, *CHOP,* and *GADD34* induced by TDP-43 overexpression was significantly decreased by Hb posttreatment (Supplementary Material Figure S2).

Taken together, these data indicate that Hb modulates ER stress induced by TDP-43, thereby mitigating neurotoxicity.

### 3.4. Hydroxocobalamin Ameliorates TDP-43-Induced Mitochondrial Dysfunction

**Figure 5:**
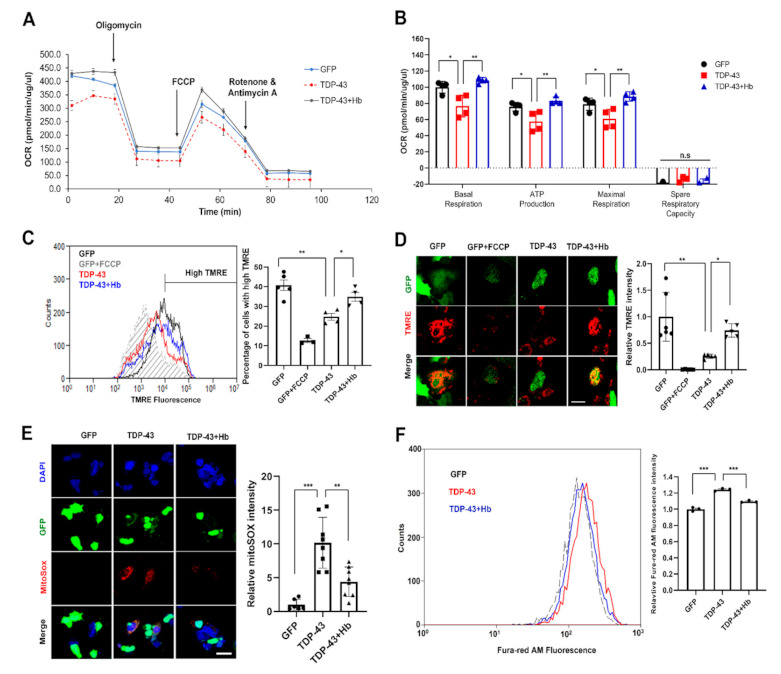
Hydroxocobalamin attenuates TDP-43-induced mitochondrial dysfunction. SH-SY5Y cells were infected with the GFP- or GFP-tagged human WT TDP-43 lentivirus and grown for 2 days. The day before harvest, cells were treated with Hb (10 µM) for 24 h. (**A**) Mitochondrial function was analyzed by detecting basal respiration, ATP production, maximal reserve and respiratory capacity with a Seahorse XF analyzer. The OCR was normalized to the total protein concentration. (**B**) Quantification of basal respiration, ATP production, maximal reserve and respiratory capacity as a percentage of basal values. Error bars represent the mean values ± SD of three independent experiments. * *p* < 0.05, ** *p* < 0.01, and n.s., not significant (one-way ANOVA with Tukey’s multiple comparison test). (**C**) Representative traces of TMRE fluorescence measured using flow cytometry (n = 3, at least 10,000 live cells were analyzed per independent replicate). The mean fluorescence intensity of GFP-tagged cells treated with the decoupling agent FCCP is indicated by the gray dotted line. The average fluorescence intensity of the high TMRE region was measured and reported as a percentage. Data are presented as the means ± SD of three independent experiments. Statistical analyses were performed using one-way ANOVA followed by Tukey’s multiple comparison test. * *p* < 0.05 and ** *p* < 0.01. (**D**) Confocal images of SH-SY5Y cells stained with TMRE. All images were collected from the Z-stack and projected as a single image. Cells were stained with TMRE (red) and GFP (green) before imaging. The ratio of TMRE-positive cells in the control or GFP-tagged human WT TDP-43-expressing cells was quantified. Quantification was performed using ImageJ software. Error bars represent the mean values ± SD of three independent experiments. The significance of differences was determined using one-way ANOVA with Bonferroni’s multiple comparison test (* *p* < 0.05 and *** *p* < 0.001). Scale bars, 20 μm. (**E**) Confocal images of SH-SY5Y cells stained with mitoSOX-red after infection with the GFP- or GFP-tagged human WT TDP-43 lentivirus and DAPI (blue) staining. Cells expressing GFP-tagged human WT TDP-43 showed significantly increased mitoSOX signals compared with the control. MitoSOX signals were restored in TDP-43-expressing cells treated with Hb. Quantification was performed using ImageJ software. Data from three independent experiments were analyzed using one-way ANOVA followed by Bonferroni’s multiple comparison test (** *p* < 0.01 and *** *p* < 0.001). Scale bars, 25 μm. (**F**) SH-SY5Y cells overexpressed GFP- or GFP-tagged human WT TDP-43, and the cells were loaded with the Ca^2+^ indicator Fura-red/AM and detected using flow cytometry. Data are presented as the means ± SD of three independent experiments. *** *p* < 0.001 (one-way ANOVA followed by Tukey’s multiple comparison test).

TDP-43 is associated with abnormal mitochondrial dynamics and dysfunction. Moreover, TDP-43 overexpression causes aberrant aggregation in mitochondria and loss of function; furthermore, WT or mutant TDP-4 impairs mitochondrial dynamics, leading to a progressive loss of neurons [63,64,65]. We examined the oxygen consumption rate (OCR), mitochondrial membrane potential, ROS production, and intracellular Ca^2+^ concentration in living cells after the induction of TDP-43 to investigate TDP-43-induced mitochondrial dysfunction (Figure 5). First, we measured the OCR using a Seahorse XF24 extracellular flux analyzer. Sequential injections of oligomycin, FCCP, rotenone, and antimycin A measured basal respiration, ATP production, maximal respiration, and spare respiratory capacity, respectively (Figure 5A). Consequently, basal respiration, ATP production, and maximum respiratory capacity were significantly reduced by TDP-43 expression, leading to mitochondrial dysfunction, and were significantly improved by Hb treatment (Figure 5A,B; basal respiration: 76.75 ± 13.42% in the TDP-43 group versus 108.63 ± 3.56% in the TDP-43 + Hb group; ATP production: 57.64 ± 12.47% in the TDP-43 group versus 83.30 ± 4.54% in the TDP-43 + Hb group; maximal respiration capacity: 61.01 ± 11.75% in the TDP-43 group versus 88.54 ± 5.90% in the TDP-43 + Hb group). In addition, spare respiratory capacity was not significantly affected by TDP-43 expression (−15.74 ± 4.47% in the TDP-43 group versus −20.09 ± 6.70% in the TDP-43 + Hb group).

Furthermore, we examined whether TDP-43 affected the mitochondrial membrane potential. GFP- and GFP-tagged human WT TDP-43-expressing cells were then analyzed using flow cytometry to quantify the fluorescence intensity of TMRE, a fluorescent dye that labels mitochondria in a membrane potential-dependent manner. The TMRE intensity was significantly lower in TDP-43-expressing cells than in controls. Moreover, Hb treatment significantly restored the mitochondrial membrane potential in TDP-43-expressing cells (Figure 5C). Consistently, similar results were obtained from confocal microscope images of living TMRE-stained cells (Figure 5D). Next, we investigated mitochondrial ROS production using mitoSOX, which is a specific mitochondrial peroxide indicator. We found that mitochondrial ROS levels increased in TDP-43-expressing cells, and Hb treatment significantly attenuated TDP-43-induced ROS production (Figure 5E). The intracellular Ca^2+^ concentration is a key regulator of mitochondrial function and functions within the organelle to stimulate ATP synthesis. However, mitochondrial Ca^2+^ overload and dysregulation are associated with several neurodegenerative diseases, including AD, PD, Huntington’s disease (HD), and ALS [66,67,68,69]. Ca^2+^ overload has been shown to contribute to necrosis or apoptosis by inducing mitochondrial dysfunction, abnormal generation of ROS, and ER stress [70]. Subsequently, we expressed TDP-43 in neuronal cells and treated them with Fura-red/AM, a fluorescent Ca^2+^-sensitive dye, for 1 h to examine intracellular Ca^2+^ homeostasis in TDP-43-expressing cells. As shown in Figure 5F, intracellular Ca^2+^ overload was observed in TDP-43-expressing neuronal cells and was significantly reduced by Hb treatment. A previous study suggested that chronic stress caused by the misfolded mutant TDP-43 protein induces inappropriate release of Ca^2+^ from the ER to the cytoplasm and eventual programmed necrosis [71]. Thus, increased Ca^2+^ concentrations in the cytoplasm drive TDP-43-mediated neurotoxicity. Taken together, these data indicate that Hb modulates cell survival by alleviating the mitochondrial dysfunction induced by TDP-43 in neuronal cells.

### 3.5. Hydroxocobalamin Regulates the TDP-43-Induced Mitochondrial Unfolded Protein Response and Mitochondrial DNA Release

**Figure 6:**
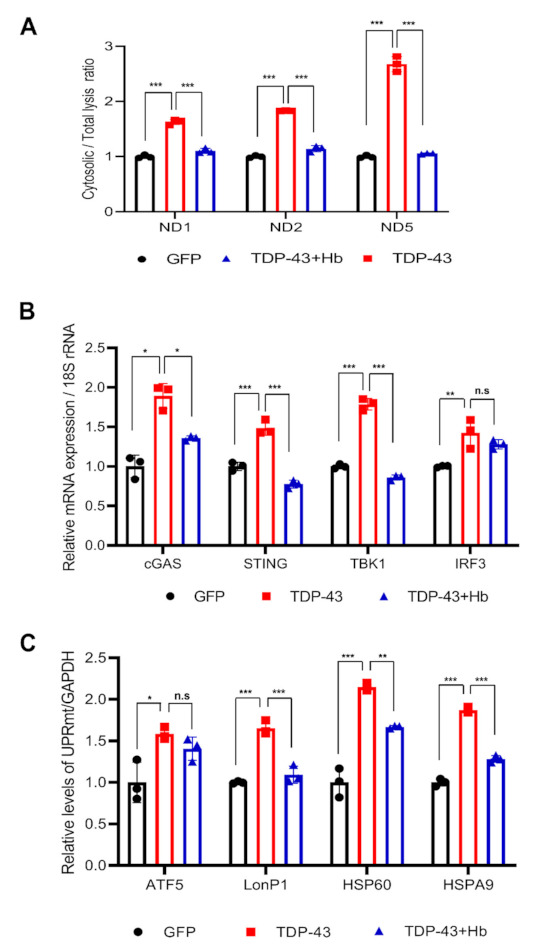
Hydroxocobalamin reduces TDP-43-induced mitochondrial DNA release and mitochondrial UPR activation. Cells expressing GFP- or GFP-tagged human WT TDP-43 were treated with Hb (10 µM) for 24 h during infection for 2 days (**A**,**B**) or 3 days (**C**). (**A**) DNA was extracted from the cytoplasmic fraction or whole cell lysate. The ratio of cytoplasmic to total mtDNA was determined using real-time PCR. Mitochondrial genes (*ND1*, *ND2*, and *ND5*) were normalized to the comparative CT value of the nuclear gene *POLG*. Values are presented as the means of individual measurements in each sample ± SD. *** *p* < 0.001 (one-way ANOVA multiple comparisons with Tukey’s post-hoc test). Using flow cytometry, only GFP-positive cells were collected and used for the mRNA analysis (**B**,**C**). (**B**) cGAS-STING pathway-related gene expression (*cGAS*, *STING*, *TBK1*, and *IRF3*) following TDP-43 overexpression in SH-SY5Y cells was measured using real-time PCR, and the expression of these genes was significantly increased by TDP-43 and reduced by Hb treatment. 18S rRNA was used for normalization. Data are presented as the means ± SD of three independent experiments. * *p* < 0.05, ** *p* < 0.01, *** *p* < 0.001, and n.s., not significant (one-way ANOVA with Tukey’s multiple comparison test). (**C**) Expression levels of mtUPR-related genes, including *ATF5*, *LonP1*, *HSP60*, and *HSPA9*, were detected using RT–PCR. *GAPDH* was used as an internal control. Error bars represent the mean values ± SD of three independent experiments. The significance of differences was determined using one-way ANOVA with Tukey’s multiple comparison test (** *p* < 0.01, *** *p* < 0.001, and n.s., not significant).

A reduced mitochondrial membrane potential and increased mitochondrial permeability regulate the release of mitochondrial DNA (mtDNA) into the cytoplasm and activate the cyclic GMP-AMP synthase (cGAS) stimulator of the interferon genes (STING) pathway [72,73]. Moreover, recent evidence indicates that the TDP-43 protein triggers mtDNA release into the cytoplasm and induces inflammation in individuals with ALS [19]. We examined whether Hb affects mtDNA release and the mitochondrial unfolded protein response (mtUPR) to understand its mechanism of altering TDP-43-induced mitochondrial dysfunction. First, GFP and GFP-tagged TDP-43-infected neuronal cells were harvested. Half of the cells were lysed to extract total DNA, and the other half was fractionated to extract cytoplasmic DNA. Then, we measured the levels of the mitochondrial genes, ND1-ND5 encoded in mitochondrial genome using RT-PCR As a result, TDP-43 increased the levels of mtDNA (ND1, ND2 and ND5) that were then released into the cytoplasm. Furthermore, Hb treatment significantly suppressed the cytoplasmic release of mtDNA (Figure 6A).

The cGAS-STING signaling pathway is known as a major mediator of inflammation in the environment of infection, cellular stress, and tissue damage [74]. Next, we sorted GFP-positive cells among GFP- and GFP-tagged TDP-43-infected neuronal cells, which were then used for analysis. We investigated the association between mitochondrial mtDNA release induced by TDP-43 expression and activation of the cGAS-STING response. TDP-43 induced the expression of cGAS, STING, and downstream factors, including TBK1 and IRF3. Hb treatment reduced cGAS, STING, and TBK1 expression induced by TDP-43 (Figure 6B).

In addition, the mtUPR is the first stress protective response initiated by mitochondrial damage, eventually mitigating damage by removing or repairing misfolded proteins [75]. We performed quantitative RT–PCR of mtUPR (*ATF5*, *LonP1*, *HSP60*, and *HSPA9*) genes in TDP-43-expressing neuronal cells. The transcription of mtUPR-related genes was upregulated by the expression of TDP-43. Subsequently, the increased expression of the LonP1, HSP60, and HSPA9 genes was reversed by Hb treatment. However, the expression of the ATF5 gene was not affected by Hb (Figure 6C). These results suggest that the induction of mtDNA release and activation of cGAS-STING signaling are essential for maintaining mitochondrial function. They may be a critical determinant of the pathogenesis of TDP-43-linked proteinopathies and may be a potential suitable therapeutic target in patients with ALS.

### 3.6. Hydroxocobalamin Mitigates TDP-43-Induced Neuronal Toxicity in Drosophila

**Figure 7:**
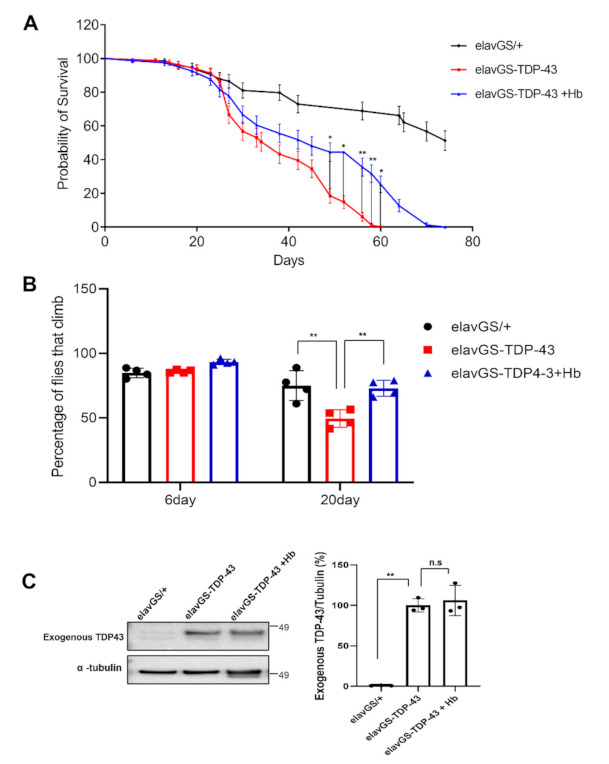
Hydroxocobalamin mitigates TDP-43-induced neuronal toxicity in a *Drosophila* model. Lifespan (**A**) and climbing ability (**B**) of elavGS-TDP-43 + DMSO or elavGS-TDP-43 + Hb flies at the indicated time points. The TDP-43-induced motility deficit was significantly rescued by treatment with Hb (10 µM). Quantification of the percentage of flies that survived and climbed. Data are presented as the means ± SEM of four independent experiments. * *p* < 0.05, ** *p* < 0.01 (one-way ANOVA followed by Tukey’s multiple comparison test). (**C**) Immunoblot analysis of TDP-43 proteins from head lysates of control or TDP-43-expressing flies. α-Tubulin was used for normalization. Data are presented as the means ± SEM. n.s., not significant (one-way ANOVA followed by Tukey’s multiple comparison test).

We next investigated whether Hb inhibits TDP-43-induced toxicity in vivo using a *Drosophila* model of TDP-43 proteinopathies, which expresses human TDP-43 in the nervous system. In our previous studies, we established a *Drosophila* model of TDP-43 proteinopathies in vivo and showed that overexpression of TDP-43 led to neurodegeneration [76,77]. We investigated the effects of Hb on the shortened lifespan and climbing defects induced by TDP-43 expression. Neuron-specific TDP-43-expressing flies showed significant defects in lifespan and climbing ability. Hb treatment prolonged the lifespan (Figure 7A). Furthermore, TDP-43-expressing flies showed a significantly reduced climbing ability compared to control animals (74.9 ± 10.0% in elavGS + DMSO versus 49.6 ± 5.8% in elavGS-TDP43 + DMSO). The TDP-43-induced climbing defect was significantly alleviated by Hb treatment (49.6 ± 5.8% in elavGS-TDP43 + DMSO versus 73.0 ± 5.3% in elavGS-TDP43 + Hb) (Figure 7B). Hb treatment alone did not affect longevity or climbing ability (data not shown). Next, we analyzed whether Hb treatment altered the level of the TDP-43 protein. No detectable change was observed in TDP-43-expressing flies treated with Hb (Figure 7C). Therefore, these results strongly suggest that Hb alleviates TDP-43-induced neurotoxicity in vivo and may be a potential therapeutic intervention for TDP-43-linked proteinopathies.

## 4. Conclusions

Vitamin B12 deficiency is associated with central nervous system lesions, including subacute combined degeneration of the spinal cord, suggesting that vitamin B12 plays an important role in the spinal cord and brain [25,78]. In addition, vitamin B12 indirectly stimulates ROS scavenging by inducing the preservation of glutathione, and thus it appears to have antioxidant properties [79]. Neurodegenerative diseases are associated with the progressive loss of specific neuronal cell populations and abnormal protein aggregates; additionally, one of the common features of these diseases is a widespread increase in oxidative stress, which may cause neuronal dysfunction or death, leading to disease pathogenesis [80,81,82,83]. Given the broad association of TDP-43 aggregation with neurodegenerative diseases, key points to determine are the factors that trigger aggregation and how TDP-43 aggregation contributes to ALS and other forms of neurodegenerative diseases. A recent study suggested that oxidative stress may be a major driving force in these disease processes [84]. However, the fundamental mechanism has not yet been fully elucidated.

Mitochondria are important organelles participating in various cellular processes, including ROS generation, ATP synthesis, and intracellular Ca^2+^ signaling. Furthermore, mitochondria are known to be key centers mediating TDP-43 cytotoxicity in the development and progression of ALS [20,85]. Neurons become critically dependent on mitochondrial function because they have a high demand for ATP production and execute complex processes of neurotransmission and plasticity. Therefore, researchers have suggested that mitochondrial dysfunction or oxidative stress play an important role in the pathogenesis of TDP-43-associated proteinopathies.

In this study, we examined whether the neurotoxicity induced by TDP-43 was alleviated by Hb treatment. Hb treatment attenuated TDP-43-induced global mitochondrial dysfunction in mammalian neuronal cells and *Drosophila* models. First, we investigated mitochondrial dysfunction upon the overexpression of TDP-43 in the human neuroblastoma cell line SH-SY5Y and confirmed that Hb treatment significantly alleviated the loss of the mitochondrial membrane potential and intracellular Ca^2+^ imbalance caused by TDP-43 expression (Figure 5). Moreover, TDP-43 significantly impaired the oxygen consumption rate in mitochondria. We also showed that TDP-43 overexpression significantly decreased both ATP production and the maximal respiratory rate in neurons. A decrease in cellular respiration and ATP production is one of the hallmarks of ALS. Hb treatment suppressed the reduced oxygen consumption rate (OCR) and mitochondrial membrane potential and increased ROS production by mitochondria in TDP-43-expressing neuronal cells (Figure 5).

Cells have a mitochondrial quality control system called the mtUPR for the repair and recovery of damaged mitochondria. mtUPR activation in mitochondria promotes cell survival and organelle repair. Recent studies have reported the role of the mtUPR in AD, PD, and ALS [18,86,87]. However, the mechanism has not yet been clearly elucidated. We found that the mtUPR, including LonP1, ATF5, HSP60, and HSPA9, was activated by TDP-43-induced mitochondrial damage, and a series of reactions were apparently initiated to restore mitochondrial protein homeostasis (Figure 6). Interestingly, the increased expression of mtUPR-related genes was significantly suppressed in Hb-treated cells. Eventually, excess accumulation of TDP-43 in the mitochondria induced irreversible mitochondrial damage (Figure 3D). Taken together, our results suggest that the activation of mtDNA release and cGAS-STING signaling is essential for maintaining mitochondrial function in TDP-43-induced proteinopathies. In addition, in the *Drosophila* disease model, the short lifespan promotion and climbing defects induced by TDP-43 expression were significantly rescued by Hb treatment. However, Hb did not affect the aggregation and mislocalization of TDP-43. Mitochondrial dysfunction, oxidative stress and UPS impairment are the major components of TDP-43-induced neurotoxicity [18,22,76,84]. Hb attenuates rotenone toxicity, which causes oxidative stress and mitochondrial dysfunction, but does not rescue MG132 toxicity, which inhibits the UPS. Thus, although Hb does not directly regulate TDP-43 pathophysiology, it appears to suppress the neurotoxicity of TDP-43 by ameliorating mitochondrial dysfunction and oxidative stress. Our findings suggest that vitamin B12 may be a potential therapeutic target for TDP-43-associated proteinopathies, and oral intake of Hb may be a potential therapeutic intervention for TDP-43-related ALS.

## Data Availability

The data are available within the article and supplementary materials.

## Supplementary Materials

The following are available online at https://www.mdpi.com/article/10.3390/antiox11010082/s1, Table S1: Primers used in qPCR experiments.Figure S1: MG132-induced toxicity was not affected by hydroxycobalamin. (A) SH-SY5Y cells were treated with various concentrations of hydroxocobalamin for 24 h, cell viability was determined using the CCK-8 assay, and the percentage of viable cells was reported as the mean ± SD of three independent experiments (n.s., not significant, one-way ANOVA using Tukey’s multiple comparison test). (B) A CCK-8 assay was performed to evaluate the effect of Hb on MG132 (2.5 µM)-induced toxicity in SH-SY5Y cells. Hb had no direct effect on MG132-induced toxicity. Data are presented as the means ± SD of three independent experiments. The significance of differences was determined by one-way ANOVA with Tukey’s multiple comparison test (n.s., not significant). Figure S2. Hydroxocobalamin mitigates the TDP-43-induced unfolded protein response in neuronal cells. Cells were infected with the GFP- or GFP-tagged human WT TDP-43 containing lentivirus and grown for 2 days. The day before harvest, the cells were treated with Hb (10 µM) for 24 h. The transcript levels of ER stress-related genes, including ATF4, CHOP, and GADD34, were detected using real-time PCR. 18S rRNA was used as an internal control. Error bars represent the mean values ± SD of three in-dependent experiments. The significance of differences was determined using one-way ANOVA with Tukey’s multiple comparison test (** *p* < 0.01 and *** *p* < 0.001).
