# Supplementary material for: Vitamin B12 Reduces TDP-43 Toxicity by Alleviating Oxidative Stress and Mitochondrial Dysfunction
Source: Antioxidants (Basel). 2021 Dec 29;11(1):82. doi: 10.3390/antiox11010082 (PMC8773243; doi:10.3390/antiox11010082)
Supplement: Supplementary file 1 [file antioxidants-11-00082-s001.zip › 2nd proofreading_ antioxidants-1460649-suppl v6.pdf]

antioxidants

MDPI

Supplementary Information

Vitamin B12 Reduces TDP-43 Toxicity by Alleviating Oxidative Stress and Mitochondrial Dysfunction

Yu-Mi Jeon [1,†], Younghwi Kwon [1,2,†], Shinrye Lee [1], Seyeon Kim [1,2], Myungjin Jo [1], Seongsoo Lee [3], Sang Ryong Kim [4,5], Kiyoung Kim [6,7,*] and Hyung-Jun Kim [1,2,*]

[1] Dementia Research Group, Korea Brain Research Institute, Daegu 41068, Korea; ekpd0345@kbri.re.kr (Y.-M.J.); yhkwon@kbri.re.kr (Y.K.); srlee@kbri.re.kr (S.L.); seyeon@kbri.re.kr (S.K.); jomj@kbri.re.kr (M.J.)
[2] Department of Brain and Cognitive Sciences, DGIST, Daegu 41068, Korea
[3] Gwangju Center, Korea Basic Science Institute, Gwangju 61886, Korea; soolee@kbsi.re.kr
[4] School of Life Sciences, Kyungpook National University, Daegu 41566, Korea; srk75@knu.ac.kr
[5] BK21 FOUR KNU Creative BioResearch Group, Kyungpook National University, Daegu 41566, Korea
[6] Department of Medical Biotechnology, Soonchunhyang University, Asan 31538, Korea
[7] Department of Medical Sciences, Soonchunhyang University, Asan 31538, Korea
* Correspondence: kiyoung2@sch.ac.kr (K.K.); kijang1@kbri.re.kr (H.-J.K.); Tel.: +82-41-413-5024 (K.K.); +82-53-980-8380 (H.-J.K.)
† These authors contributed equally to this work.

Supplementary Methods

Supplemental Table S1. Primers used in qPCR experiments.

Gene	Accession number	Direction	Primer sequence	Species
ATF4	NM_001675	Forward	5′-GAGGTGGCCAAGCACTTCAA	*H. sapiens*
		Reverse	5′-GCCCGCCTTAGCCTTGTC	
CHOP	NM_001675	Forward	5′-GGGAGCTGGAAGCCTGGTAT	
		Reverse	5′-CCCCCATTTTCATCTGAAGACA	
GADD34	U83981	Forward	5′-CCCAGAAACCCCTACTCATGATC	
		Reverse	5′-TCGGAGAAGCGCACCTTT	
ND1	LC178901	Forward	5′-CCCTTCGCTGACGCCATA	
		Reverse	5′-TGGTAGATGTGGCGGGTTTT	
ND2	AF014899	Forward	5′-GCAAGCAACCGCATCCATA	
		Reverse	5′-CATTGTCCGGAGAGTATATTGTTGA	
ND3	JN034128	Forward	5′-CCTACCATGAGCCCTACAAACAAC	
		Reverse	5′-GGTGATGATTAATAAGAGGGATGACA	
ND4	JN034131	Forward	5′-AGCTCCATCTGCCTACGACAA	
		Reverse	5′-TATGTGGCTGATTGAAGAGTATGCA	
ND5	DQ926980	Forward	5′-CACAGCAGCCATTCAAGCAA	
		Reverse	5′-AGGCGAGGATGAAACCGATA	
POLG	NM_002693	Forward	5′-AAGCAGGGCAAACACAAGGT	
		Reverse	5′-GGCTTTCCTCTGGGACTTCTG	
cGAS	KC294566	Forward	5′-CGGGCGGTTTTGGAGAA	
		Reverse	5′-GCCGCCGTGGAGATATCA	
STING	NM_198282	Forward	5′-CTGGCCCCAGCTGAGATCT	
		Reverse	5′-CATGGGCCACGTTGAAATTC	
TBK1	XM_005268809	Forward	5′-TGGAAGGCTGGCACAACAT	
		Reverse	5′-GAGGTTCCCGGCTTACTACAAA	
IRF3	NM_001571	Forward	5′-TAAGCCAGACCTGCCAACCT	
		Reverse	5′-CTGCTAAACGCAACCCTTCTTT	
ATF5	NM_012068	Forward	5′-GGACCGCAAGCAAAAGAAGA	
		Reverse	5′-CGCTGGCGGTACCTCAGA	
LonP1	XM_011528441	Forward	5′-CCAACGTCACGGACACCAT	
		Reverse	5′-CGACACGTTGATCATCTCCATAC	
HSP60	AF143723	Forward	5′-GGCGGCCATCGGAGTT	
		Reverse	5′-ATCCTTATAGACGGCCTCACATG	
HSPA9	NM_004134	Forward	5′-TTCTTGTGGGTGGCATGACTAG	
		Reverse	5′-TGCCAAAAAGATCCTGTACAGTCT	
18S rRNA	M10098	Forward	5′-CGGCGACGACCCATTC	
		Reverse	5′-CACGGCGACTACCATCGAA	
GAPDH	NM_002046	Forward	5′-GTCAGTGGTGGACCTGACCT	
		Reverse	5′-TGCTGTAGCCAAATTCGT TG	

Supplementary Figure

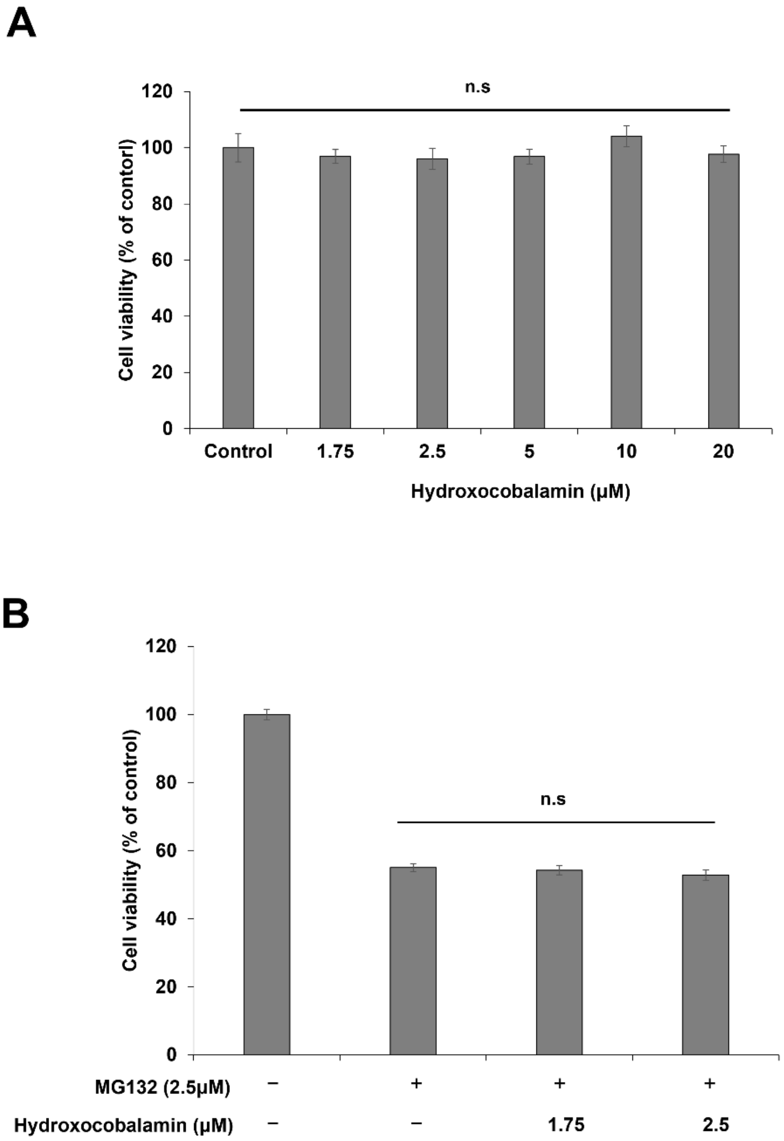

Figure S1. MG132-induced toxicity was not affected by hydroxycobalamin. (A) SH-SY5Y cells were treated with various concentrations of hydroxocobalamin for 24 h, cell viability was determined by CCK-8 assay, and the percentage of cell viability was expressed as the mean ± SD of three independent experiments (n.s., not significant, one-way ANOVA using Tukey's multiple comparison test). (B) A CCK-8 assay was performed to evaluate the effect of Hb on MG132 (2.5 μM)-induced toxicity in SH-SY5Y cells. Hb had no effect on MG132-induced toxicity. Data are presented as the mean ± SD of three independent experiments. The significance was determined by one-way ANOVA with Tukey's multiple comparison test (n.s., not significant).

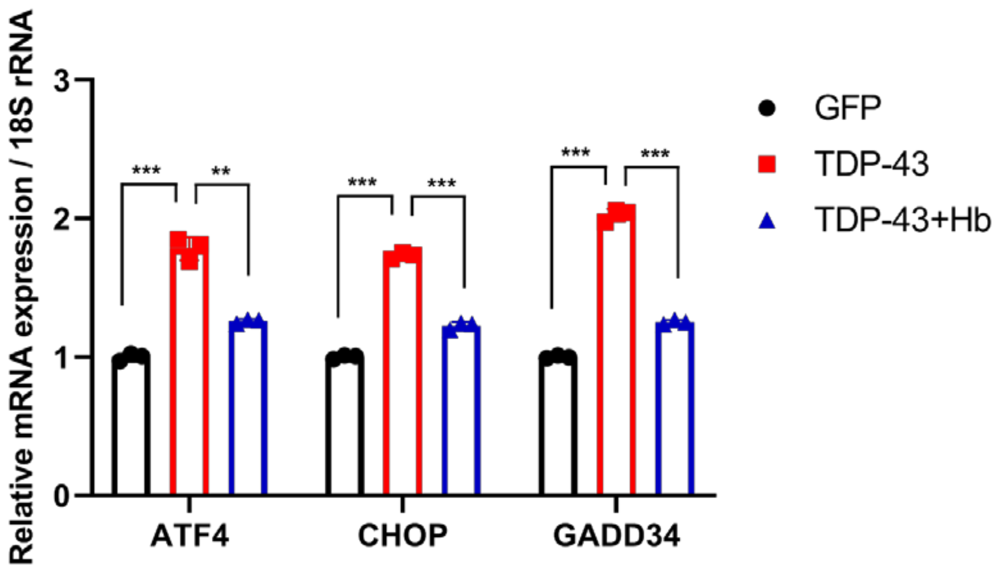

Figure S2. Hydroxocobalamin mitigates the TDP-43-induced unfolded protein response in neuronal cells. Cells were infected with the GFP- or GFP-tagged human WT TDP-43 containing lentivirus and grown for 2 days. The day before harvest, the cells were treated with Hb (10 μM) for 24 h. The transcription levels of ER stress-related genes, including *ATF4*, *CHOP*, and *GADD34*, were detected using real-time PCR. 18S rRNA was used as an internal control. Error bars represent the mean values ± SD of three independent experiments. The significance of differences was determined using one-way ANOVA with Tukey's multiple comparison test (**$p < 0.01$ and ***$p < 0.001$).
